# Dance Training Shapes Action Perception and Its Neural Implementation within the Young and Older Adult Brain

**DOI:** 10.1155/2018/5459106

**Published:** 2018-07-12

**Authors:** Louise P. Kirsch, Nadine Diersch, Dilini K. Sumanapala, Emily S. Cross

**Affiliations:** ^1^Social Brain in Action Laboratory, Wales Institute for Cognitive Neuroscience, School of Psychology, Bangor University, Bangor, UK; ^2^Research Department of Clinical, Educational, and Health Psychology, Division of Psychology and Language Sciences, Faculty of Brain Sciences, University College London, London, UK; ^3^Aging & Cognition Research Group, German Center for Neurodegenerative Diseases (DZNE), Magdeburg, Germany; ^4^Centre for Brain and Cognitive Development, Birkbeck College, University of London, London, UK; ^5^Institute of Neuroscience and Psychology & School of Psychology, University of Glasgow, Glasgow, UK

## Abstract

How we perceive others in action is shaped by our prior experience. Many factors influence brain responses when observing others in action, including training in a particular physical skill, such as sport or dance, and also general development and aging processes. Here, we investigate how learning a complex motor skill shapes neural and behavioural responses among a dance-naïve sample of 20 young and 19 older adults. Across four days, participants physically rehearsed one set of dance sequences, observed a second set, and a third set remained untrained. Functional MRI was obtained prior to and immediately following training. Participants' behavioural performance on motor and visual tasks improved across the training period, with younger adults showing steeper performance gains than older adults. At the brain level, both age groups demonstrated decreased sensorimotor cortical engagement after physical training, with younger adults showing more pronounced decreases in inferior parietal activity compared to older adults. Neural decoding results demonstrate that among both age groups, visual and motor regions contain experience-specific representations of new motor learning. By combining behavioural measures of performance with univariate and multivariate measures of brain activity, we can start to build a more complete picture of age-related changes in experience-dependent plasticity.

## 1. Introduction

Throughout the lifespan, when learning a new skill such as riding a bicycle or dancing the tango, we benefit from not only physically practicing the new skill but also from watching others who can already perform that skill. Our ability to learn by physical practice as well as by observation is a key ingredient for acquiring new motor skills and is thus essential for us to survive and thrive within a social world. For over a century, it has been suggested that actions learned by physical or observational practice are represented within common cognitive and neural structures [1]. However, behavioural and brain-based investigations to date have not satisfactorily examined the extent to which this is actually the case. Moreover, how the aging process impacts our ability to learn via physical practice and observation remain underexplored. Such a lack of knowledge means that critical questions for understanding how best to facilitate new learning in educational and therapeutic contexts are ripe for exploration.

Prior work demonstrates the existence of an action observation network (AON), comprising sensorimotor brain regions including premotor, parietal, and occipitotemporal cortices [2–4]. These brain regions have been shown to be engaged when watching others in action and respond more robustly when observing actions that have been physically practiced [5–9] or visually experienced [10–14], compared with similar actions with which participants have had no prior experience.

Since the original work establishing the functionality of the action observation network, a rich literature examining how laboratory-based complex action training interventions shape the relationship between action and perception has emerged [5, 15–22]. As mentioned above, what many of these studies demonstrate is that the more familiar an action is, the stronger the response is within core AON regions [5, 6, 18, 23, 24]. On the other hand, an increasing number of studies report findings demonstrating that AON activity does not *necessarily* follow this linear trend of increasing engagement with increasing familiarity [17, 25–28]. These studies demonstrate equivalent or greater AON activity when participants observe *less* familiar actions (compared to more familiar actions), which is often interpreted as increases in neural efficiency [29–32].

Such marked differences in how the response amplitude of sensorimotor cortices changes after learning have been well documented in the motor control literature more broadly (see [33–35] for reviews). This has led to the suggestion that human neuroimaging investigations of sensorimotor learning are limited by the blunted sensitivity of traditional magnitude-based blood oxygen level-dependent (BOLD) approaches [36, 37]. An increasing number of studies are moving beyond univariate approaches of examining sensorimotor learning in the human brain, by attempting to evaluate *representations* of learning, as evidenced by more subtle modulations of the voxel-by-voxel activity patterns within the same area of cortex (e.g., [38, 39]). The ability to more closely evaluate learning representations is made possible using multivoxel pattern analyses (MVPA), an analytic approach we use in the present study to not only address how complex action learning shapes engagement of sensorimotor cortices but also how the same complex action training paradigm shapes brain responses within both young and older adults. Through combining training interventions with the increased analytical sophistication of MVPA, the present study explores how physical and observational learning impacts behaviour and the corresponding neural representations of actions.

With advancing age, sensorimotor and cognitive resources needed for learning a new motor skill decline and the efficacy of the matching process between observed and executed actions appear to be compromised [40]. However, this is modulated by the type of action observed. Evidence from studies investigating age-related changes in biological motion perception, motor imagery, and action observation suggests that forming an internal action representation may remain relatively preserved with advancing age for simple movement sequences, whereas it appears to become more imprecise in conditions with higher task complexity or when flexible adaptations to changes in the environment are required [41–43]. Such changes in behavioral performance are typically accompanied by a loss of neural selectivity in relevant regions of the aging brain [44, 45]. Overactivations, particularly in sensory cortices, are frequently reported leading to the assumption that older adults might be less adept at embodying new actions compared to younger adults [46, 47]. Thus, their ability to form an action representation based on physical and observational training might be limited, particularly compared to younger adults.

However, it remains unknown how learning complex whole-body actions, such as those that might be required in a fitness or social dance class, shapes behavioural and brain responses in young compared to older adults. In the present study, we aimed to explore this question by manipulating the type of sensorimotor experience that young and older adults received with dance sequences to determine how experience shapes responses at behavioural and brain levels. We attempt to address three main questions: (i) How do different types of training influence complex action performance among young compared to older adults? (ii) How do different types of training shape brain responses at a global level during action observation among young compared to older adults? (iii) How does physical training shape brain responses at the level of action representations? To address the first two questions, we implemented similar behavioural and univariate functional magnetic neuroimaging (fMRI) procedures reported in previous training studies [3, 18, 48] and studies that have compared the impact of complex action experience among young and older adults [43, 49]. To address the third aim, we used multivariate decoding procedures [50] to determine whether distributed voxel activity could be used to discriminate between patterns associated with viewing physically trained and untrained movements in the brains of young compared to older adult participants.

## 2. Methods

### 2.1. Participants

Twenty-three physically and neurologically healthy young adults were recruited from the Bangor University student population, and nineteen physically and neurologically healthy older adults were recruited from the local community. Older adults were screened for any past medical history, and we excluded any participants who reported any prior neurological diseases or use of medication that might alter their performance during the task or fMRI scanning. In addition, they were asked to complete the MMSE [51] to assess any cognitive impairment (M = 28.9, range = 26.5–30.0, maximum score: 30) and an fMRI safety screening questionnaire. The older adult participants were also invited to perform one dance sequence from the Dance Central Kinect Game before the study began, to ensure they felt comfortable with the technical equipment and were able to take part in all the procedures that would be required in the full study. Only those older adults who enjoyed this experience and were eager to take part after this prestudy trial were selected to take part. All participants (young and older adults) were dance naïve, meaning they had limited or no experience performing or observing dance, and none reported prior experience playing dance video games. All participants were right-handed or ambidextrous (ranging from moderately right-handed to strong right-handed; young adults M = 60.78, SD = 22.20; older adults M = 86.58, SD = 20.91, range 33–100), as assessed by the Edinburgh Handedness Inventory [52]. Two younger adult participants were excluded from the final sample due to excessive head motion artefacts whilst undergoing fMRI scanning, and one younger adult dropped out of the study half way through the training phase and thus was excluded due to having an incomplete dataset. The final sample comprised 20 younger participants (12 females) with a mean age of 19.5 years (SD = 1.54 years, range 18–23 years) and 19 older participants (11 females) with a mean age of 63.6 years (SD = 4.4 years, range 55–69 years). All participants provided a written informed consent prior to taking part in any study procedures and were reimbursed for their involvement with either cash or course credit. The Bangor University School of Psychology Research Ethics Committee approved all components of this study (protocol number 2014-13123-A12806).

### 2.2. Stimuli and Apparatus

**Figure 1:**
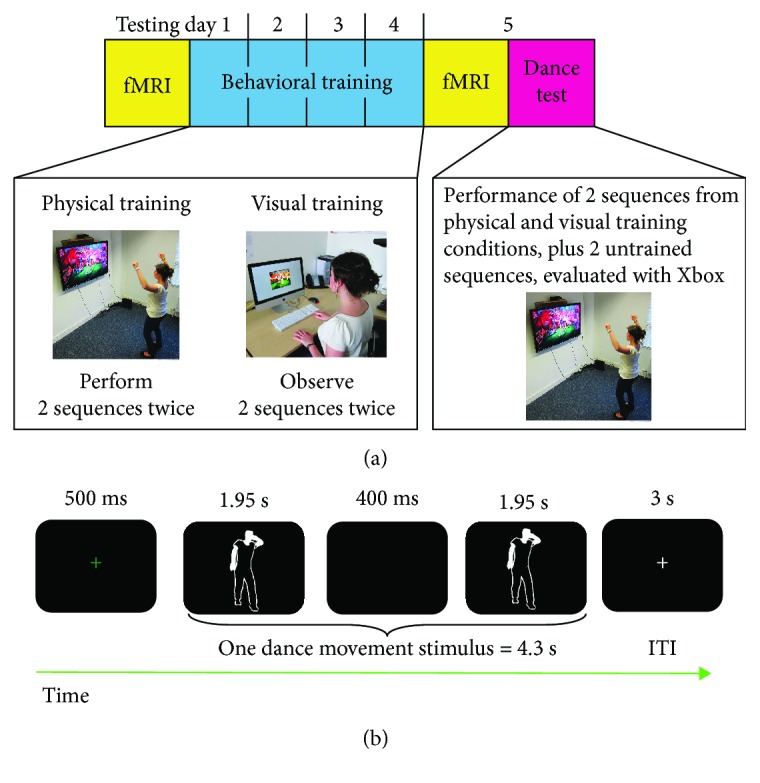
(a) Overview of experimental procedures. Participants of both age group underwent the exact same procedure. First they underwent a scanning session on day 1 and then started the behavioural training on that same day and the following 3 days. During each training day (days 1–4), participants physically trained with 2 long sequences and visually trained with other long sequences. Two other long sequences remained untrained. On day 5, participants underwent the same scanning session as on day 1. After scanning on day 5, participants had to physically perform all the long sequences (physically, visually trained, and untrained). (b) Schematic of an example fMRI trial. Each typical fMRI trial consisted of a 500 ms fixation cross, one dance movement stimulus lasting for 4.3 s, and an intertrial interval (ITI) of 3 s.

Six dance sequences from the dance game “Dance Central 2” (Harmonix Music Systems, 2011) for the Xbox 360 Kinect™ console were chosen that featured gender-neutral dance movements. The six chosen dance sequences were specifically selected so as to contain no overlapping dance moves between songs (i.e., each move was uniquely associated to one song/dance sequence). Each dance sequence was set to a popular song (e.g., *Like a G6* by Far East Movement or *What is love* by Haddaway) and varied in length from 2:20 to 2:29 minutes (average length = 2.22 s; SD = 10 s) and in tempo between 105 and 129 bpm (average tempo = 118.83 bpm; SD = 11.21). To focus participants' attention on the avatar whose moves they were learning, the same background setting was selected for all dance videos, which had a minimal amount of extraneous movement. The difficulty of the dance sequences (complexity and amplitude of dance movements) was set to a minimum level to ensure participants across both age groups could perform them to some degree from the very first training day but would still have ample room for improvement. The six dance sequences were paired to create three groups whose composition was matched for number and complexity of specific dance movements, as well as tempo. Each pair of sequences was assigned to one of the three training conditions: physical training, visual training, and no experience/untrained (Figure 1). A total of three different training groups were assembled, meaning that each pair of dance sequences was trained in all three training conditions across participants.

Animated silhouettes from the game depicting individual movements from the preselected dance sequences were captured and used as stimuli during both pre- and post-training fMRI sessions. The use of silhouettes, instead of original game footage, was specifically chosen to reduce visual cues associated with the original training context and to focus attention on the movements alone [48]. In this way, brain activity recorded when observing these pared-down dance movements should be more attributable to sensorimotor experience. 18 short animated silhouettes dance segments without music were extracted using iMovie ‘11 (Apple Inc.) and edited using Adobe Premiere Pro (Version 7.1 for Microsoft Windows 7), three sequences from each full dance sequence. The resultant 18 stimuli were matched for length to all be 1.95 seconds. Each stimulus was edited so that it featured one complete, coherent dance move involving whole-body motion and significant spatial displacement of the limbs (cf. [53]). All stimuli were novel to the participants during the pre-training fMRI scan.

### 2.3. Behavioural Training Procedure and Analysis

Both age groups underwent identical training and testing procedures. Participants were randomly assigned to one of three training groups in which they experienced the same pairs of sequences assigned to the two training conditions (place between the pre- and post-training fMRI scanning sessions) (Figure 1). For each training session, participants completed physical and visual training on the set of sequences to which they had been randomly assigned. Participants physically practiced the same two sequences twice (once with a female and once with a male avatar) and observed two different sequences twice. The order in which participants completed the training conditions was counterbalanced within and between participants across training days. Each training session lasted approximately 30 minutes.

#### 2.3.1. Physical Training

For sequences of which participants physically practiced, they stood approximately 2 meters away from a 52^″^ Sharp flat screen television mounted on the wall in front of them. Participants' task was to mirror the dance movements of the avatar in the *Dance Central 2* Xbox 360 game as closely as possible and concentrate on improving their performance during subsequent sessions. The Kinect motion capture system compared participants' movements to the avatar's movements and assigned a score based on accuracy of mirroring the avatar. The Kinect‘s scoring system is based on how closely participants match the temporal and spatial features of the avatar's movements, including the avatar's movement amplitude. As the Kinect is a closed system consumer product, further details about how scores are assigned are not available. Similar procedures using this system were successfully applied in previous studies measuring the neural effects of dance training in young adults (see also [18, 48, 54, 55]). The game provides on-screen feedback about performance accuracy in the form of a final score after each sequence. However, to make the physical training condition as comparable as possible to the visual training condition (where no performance feedback was given), we covered the side margin of the TV screen (where the score is displayed after performance) so that participants were not aware of their dance scores after performing each sequence. These participant dance scores were recorded by the researcher and used as an objective measure of dance performance ability for the behavioural analyses.

The four overall dance scores participants received each day for the dance sequences in the physical training condition were averaged so that each participant had a single score representing dance performance for each training day. A mixed ANOVA with training day assigned as a within-subjects factor with four levels (training days 1–4), and age group as between-subject factor (young adults, older adults) was conducted on these scores in order to determine how performance across consecutive days of training compared between age groups. Additionally, we performed a repeated measure ANOVA for each age group separately to confirm the training manipulation worked and that physical performance increased across the daily training sessions.

#### 2.3.2. Visual Training

For the sequences for which participants acquired visual experience, they sat comfortably in front of a computer running Psychophysics Toolbox 3 in MATLAB R2010a (MathWorks Inc.), which presented the full dance videos. Each video was shown twice, once for each avatar (male, female), in a random order. The dimensions of the dance videos were 640 × 480 mm, which reflected perceptually similar scaling to the physical training condition. As well as visual information, participants listened to the soundtrack that accompanied each sequence via the computer speakers. Participants were instructed to pay close attention to the dance sequences and were told that they would have to perform the sequences at the end of the week, so they should try to learn the movements as best as they could. To test that they were paying close attention, at the end of each music video, ten short dance segments (five from the videos they had just watched) were displayed, without music, each followed by the question “Did you see this movement in the video you just watched?”. Participants had to respond “yes” or “no” using the keyboard arrow keys. All test videos were presented silently (as the task would have been too easy if the accompanying soundtracks were also presented).

An accuracy score for each participant for each of the four days of training was calculated based on their performance on this task. Similar analyses done for the physical dance scores were performed on the visual accuracy scores.

#### 2.3.3. Post-Training Performance Assessment

On the final day of the study (day 5), participants returned to the laboratory to perform the four full dance sequences used in training (two physically trained sequences and two visually trained sequences) as well as the two untrained sequences (segments that they had observed during both fMRI sessions only). The test followed the same paradigm as the physical training phase of the study: participants physically performed the dance sequences from all six songs, mirroring the avatar's dance movements as closely as possible whilst the Kinect system captured and scored their movements. The six sequences were randomised and balanced for the gender of the avatar. Objective performance scores were obtained in the same way as for the physical training condition.

Raw scores from both exemplars from each training category were averaged within training conditions to produce an average score per participant for each of the three test conditions. We first performed a mixed-design ANOVA using an age group as a between-groups factor to compare dance performance between young and older adults on day 5. To further investigate performance in each age group independently, we next performed repeated-measures ANOVAs on these scores to investigate the impact of different kinds of experience on physical performance. Pairwise comparisons (Bonferroni corrected for multiple comparisons, with adjusted alpha levels of 0.025) were subsequently evaluated to investigate differences between conditions in more detail. Degrees of freedom reflect the Greenhouse-Geisser correction where sphericity has been violated.

#### 2.3.4. Training Modality Categorization Task

On day 5, immediately following the post-training fMRI scan and before the post-training performance assessment, participants performed a short control task, similar to the one in Sumanapala et al. [48]. In this task, participants watched again each dancing silhouette stimulus they observed during scanning (18 in total; 6 per training category). After each stimulus, participants were asked to categorize the movement as being either “physically trained,” “visually trained,” or “untrained”, using the 1, 2, and 3 keys on a computer keyboard. Answers were untimed. Accuracy scores for each training condition were computed for each age group.

### 2.4. Neuroimaging Procedure

Each participant completed one fMRI session prior to the training procedures and an identical session immediately following the four days of training (Figure 1(a)). Participants completed 6 runs within each scanning session, lasting an average of 9 min and containing 60 trials each. In each run, participants watched three times 18 stimuli featuring short dance segments taken from the three training conditions (physically trained, visually trained, and untrained; 6 stimuli per training condition). Unlike the video footage used during training, the videos used during scanning featured the silhouette of an avatar performing each dance movement, which lasted 1.95 seconds. Each individual dance movement was presented twice in a row with a 400 ms black screen between each presentation (see Figure 1(b)). Each stimulus was preceded by a green fixation cross presented for 500 milliseconds, to announce the next trial. Each dance stimulus was followed by a fixation cross presented for a fixed duration of 3 seconds. After this, the next trial started. Finally, six additional video stimuli (featuring dance movements that were not part of the full set of 18 videos taken from the training conditions—these dance movements were never encountered outside of scanning) were included for attentional control questions. After each of these six test trials, participants were asked a question that required a yes or no response (button responses were counterbalanced across participants, with an index finger press corresponding to a yes response and a middle finger press corresponding to a no response for half of the participants and the inverse response schedule for the other half of participants). Participants had 4 seconds to provide a response via a four-button fibre optic response box placed on their lap on which they rested the index finger and middle fingers of both hands over the buttons. The question that appeared was randomly selected to be one of the following four: “Did the dancer place at least one arm above his head?,” “Did the dancer reproduced the same movement on the left and on the right?,” “Did the dancer take a step forward?,” or “Did the dancer move his legs?.” These questions appeared in a random order and were designed to ensure participants paid full attention to the dancer's movement in each stimulus. Each test trial was followed by a 12-second fixation cross that served as implicit baseline. Participants were familiarized outside the scanner prior to the pre-training scan with all features of the experiment and what they would be asked to do whilst in the scanner.

Stimulus presentation and response recording was done via a Mac desktop computer running MATLAB R2013a (MathWorks, Natick, MA) and Psychophysics Toolbox 3 [56–58]. The video stimuli were presented on a 24^″^ LCD BOLDscreen (Cambridge Research Systems), which was visible to participants via a mirror mounted on the head coil. The experiment was carried out in a 3 T Philips MRI scanner using a SENSE phased-array 32-channel head coil. For functional imaging, a single-shot echo planar imaging (EPI) sequence was used (T2∗-weighted, gradient echo sequence; echo time TE = 30 ms; flip angle, 90°). The scanning parameters were set as follows: repetition time TR = 2500 ms; 38 transverse slices; voxel dimensions, 2.3 × 2.3 mm with voxel slice thickness = 3 mm; slice gap = 0.1 mm; field of view, 224 × 224 × 118 mm; matrix size, 96 × 95 mm × 38 slices; and anterior-posterior phase encoding. Parameters for T1-weighted anatomical scans were 240 × 224 × 175 mm; voxel dimensions, 1 × 1 × 1 mm; TR = 12 ms; TE = 3.5 ms; and flip angle = 8°. All the scans were collected in an ascending order. For each run of each scanning session, the first two brain volumes were discarded to reduce saturation effects. 224 volumes per functional run were collected for each participant.

### 2.5. fMRI Data Analysis

#### 2.5.1. Univariate Analyses

Neuroimaging data from each scanning session (before and after training) were preprocessed together to facilitate the construction of first-level design matrices including data from both scanning sessions. When there are several sessions, data can be either preprocessed separately or together. We decided to combine neuroimaging data from both scan sessions at the first level of analyses, for several reasons: (i) this leads to both days sharing the same implicit baseline, subsequently reducing the likelihood of results emerging simply due to differences between the two scanning sessions that are not result of the training manipulation, per se; (ii) the smoothness estimation on the data should be better with more data points, thereby reducing the threshold for multiple comparison correction using random field theory; and (iii) this preprocessing method was necessary for the subsequent RSA analyses.

Using SPM12 (Wellcome Department of Imaging Neuroscience, London, UK), data were realigned and unwarped, coregistered to the individual participants' T1 scans, and normalized to the Montreal Neurological Institute (MNI) template. Slice timing correction was performed after realignment, and all images were finally spatially smoothed using an 8 mm FWHM Gaussian kernel. A design matrix was fitted for each participant with a high-pass filter cut-off of 128 s, with each type of dance video (physical training, visual training, and untrained conditions), as well as attentional control videos and button presses associated, modelled together as a boxcar function convolved with the hemodynamic response function with temporal and dispersion derivatives. Additionally, participant-specific movement parameters were modelled as separate regressors of no interest.

The univariate analyses were designed to achieve three aims:

- To test both direct matching and neural efficiency accounts of experience-dependent plasticity, we first examined both increases and decreases in training-related brain activity among young and older adults separately. The most rigorous test of these effects involves evaluating scanning session by training experience interactions (i.e., to assess physical training experience, e.g., this would involve contrasting physical training > untrained on the post-training scan session compared to the pre-training scan session and the inverse (e.g., [18, 27, 59])). However, when evaluating these analyses in both directions, for physical and visual training experience separately, and among young and older adults independently, no brain regions survived a *p* < 0.05_FWEcorrected_ threshold. This is not necessarily a cause for concern, as the type of manipulation and stimuli we have used in the present study could reasonably be expected to have subtle effects that do not meet the most stringent imaging threshold criteria (see, e.g., [3, 16]).
- In order to probe more subtle influences of our training manipulation and visual task in the scanner, we also evaluate direct contrasts comparing each training condition to itself in the pre- and post-training scans. We therefore examined brain regions that showed either increases or decreases in BOLD responses after compared to before training, for physical and visual training separately. These analyses were performed at the whole brain level while focusing on activations that survived a FWE-corrected threshold of *p* < 0.001 at the cluster level. This was achieved by contrasting pre- and post-training brain activity separately for dancing silhouettes that were associated with physical training or visual training at the 1st level for each participant and then conducting one-sample *t*-tests at the group level. The identical procedures were repeated for the young and older adults' data.
- The second set of univariate analyses directly compared neural responses between young and older adults from the physical training condition. We focused on this training condition in particular as this was the most intensive type of training where we would reasonably expect the most robust effects to emerge [16, 18, 39]. This was achieved by using a two-sample *t*-test at the group level, which compared the 1st-level contrasts evaluating physical training before and after training, among young adults compared to older adults. To avoid any contamination of brain findings due to differences in physical performance scores/abilities between young and older adults, physical performance gains (calculated as the difference between physical performance scores on day 5 − day 1) were included as a covariate.
- Finally, similarities between young and older adults when observing sequences that have been physically trained were examined through a customised conjunction analysis based on that reported in [18]. This analysis examined brain regions that showed increases or decreases after four days of physical training when both young adults and older adults observed these sequences after physical training compared to pre-training.

#### 2.5.2. Multivariate Analyses

After evaluating magnitude-based univariate analyses, we next performed multivariate analyses to ascertain with finer detail on how sensorimotor training experience shapes neural representations during action observation among young and older adults. Specifically, this approach enabled us to explore how young and older brains distinguish between different types of training experience. Using The Decoding Toolbox scripts [50], we performed whole-brain searchlight decoding to assess the degree of outcome adaptation in local fMRI patterns surrounding each voxel (radius 8 mm) for each participant using the unsmoothed, realigned, and normalized imaging data.

This procedure involves extracting voxel pattern information from individual subject beta values generated during first-level preprocessing within SPM. Pattern information is specifically extracted from these beta values using support vector machine (SVM) algorithms that maximise mathematically defined representational distances within a shared coordinate space between different classes of data [50, 60]. To achieve this distinction, a data set is usually split into “training sets” and “test sets” before being introduced to SVM pattern recognition algorithms for classification. The algorithms are trained to identify patterns of data associated with specific classes within a training set before being tested on their ability to identify class membership on an unknown “test” set. Within neuroimaging, training sets and test sets can be shuffled in a leave-one-out cross-validation procedure [61], which limits the likelihood that spurious noise within specific subsets of data may lead to biases in pattern classification accuracy.

Following this, we implemented GLM analyses similar to the ones described for the univariate analyses. This involved modelling each training condition separately (physical, visual, and untrained) as well as attentional control videos and button presses. Also included in this model were participant-specific movement parameters, modelled as separate regressors of no interest. Physical and untrained events served as inputs for the classifiers and were labelled according to the model. Data were then split into different training and testing subsets depending on the model, using in turn each separate run as training and test runs [62]. Classification accuracy values (corresponding to observed prediction accuracy minus chance prediction for each voxel, with chance being 50%) for each analysis were entered into second-level *t*-tests for group-level analysis on a voxel-by-voxel basis. We decided to take “to be physically trained” and “to remain untrained” sequences as input for the classifiers, as previous literature [17, 39] documents that the most robust differences emerge after physical training, a finding corroborated by the univariate analyses from this study as well.

The multivariate analyses were designed to achieve two distinct aims:

- The first set of analyses is aimed at identifying brain regions that can distinguish between physical and untrained movement sequences after training compared to before training, among young and older adults separately. Naturally, we would predict this between-category classification accuracy to be better overall after training compared to before training, when all stimuli were equally unfamiliar. This was achieved by running the searchlight decoding scripts on the 1st-level data from pre-training and post-training scans separately, taking as the two classifiers the “physically trained” movement sequences and the “untrained” movement sequences, and then running paired-sample *t*-tests at the group level, separately for young and older adults.
- The aim of the second set of analyses was to compare classification accuracies between the two age groups. We were particularly interested in accuracy to distinguish between physically trained and untrained sequences after physical training (which entails examination of the post-training scan data only). To achieve this, we first ran a separate whole-brain decoding analysis to determine which brain regions distinguish between physically trained and untrained sequences after training, separately for each age group. The results of this analysis for both age groups are presented in Supplementary Table 1. Then, contrasts from post-training classification for young and older adults were entered into a two-sample *t*-test. Finally, we explored which regions in young and older adults could similarly distinguish between physically trained and untrained movements after training by running a conjunction analysis, similarly to the one done for the univariate analysis above (and in [18]).

## 3. Results

### 3.1. Behavioural Results

#### 3.1.1. Physical and Visual Training

**Figure 2:**
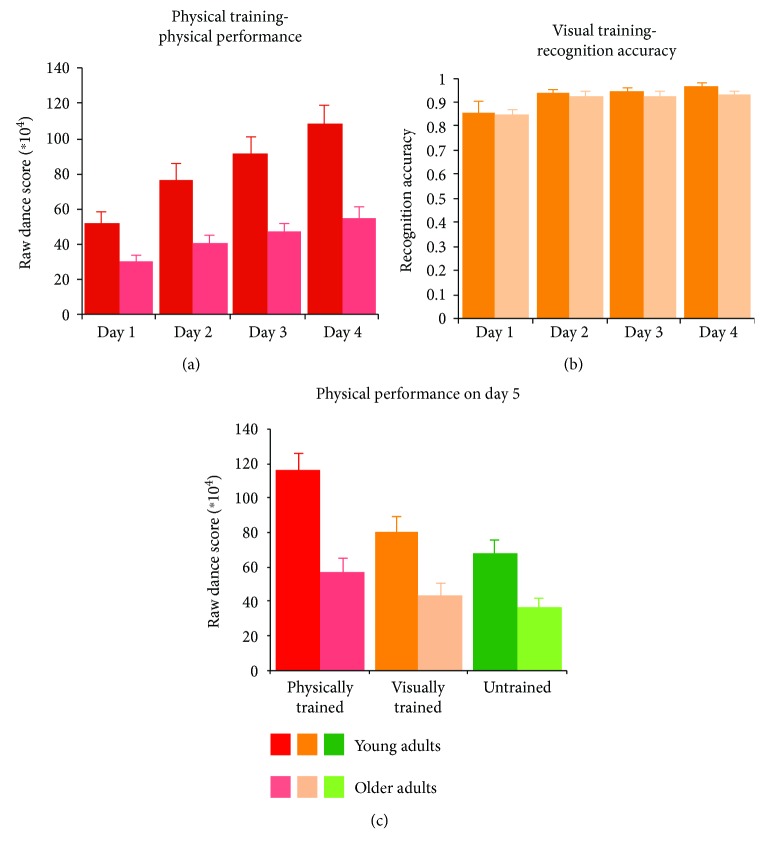
Training performances for both age groups. (a) Physical performance across the 4 consecutive days of physical training, for both younger (bright red) and older adults (pale red). (b) Recognition accuracy after each day of visual training, for both younger (bright orange) and older adults (pale orange). (c) Average physical performance on day 5, for each training condition (physical, visual, and untrained sequences), for both younger (darker bars) and older adults (lighter bars).

For each participant, physical performance was assessed each day by averaging performance scores across both dance sequences assigned to the physical training condition (Figure 2(a)). When evaluating young and older adults' physical performance across days of training, a main effect of training day was observed (*F*_(2.086, 75.088)_ = 79.476, *p* < 0.001, *η*_p_^2^ = 0.688) as well as an interaction between age group and performance across days, with younger adults performing better than older adults (*F*_(2.086, 75.088)_ = 14.320, *p* < 0.001, *η*_p_^2^ = 0.285). In both age groups, a main effect of training day was observed whereby physical performance significantly improved across the four days of training (young adults: *F*_(2.026, 36.47)_ = 50.218, *p* < 0.001, *η*_p_^2^ = 0.736; older adults: *F*_(3, 54)_ = 33.417, *p* < 0.001, *η*_p_^2^ = 0.748). Pairwise comparisons indicate that significant differences were observed between all possible pairs of days (young adults: day 1 versus day 2: *t*_(18)_ = −5.430, *p* < 0.001; day 2 versus day 3: *t*_(18)_ = −3.960, *p* = 0.001; and day 3 versus day 4: *t*_(18)_ = −3.375, *p* = 0.003; older adults: day 1 versus day 2: *t*_(18)_ = −5.225, *p* < 0.001; day 2 versus day 3: *t*_(18)_ = −2.981, *p* = 0.008; and day 3 versus day 4: *t*_(19)_ = −3.375, *p* = 0.003).

Participants' performance on the visual training task was assessed through a movement recognition task after observing two different sequences (Figure 2(b)). A main effect of visual training was found across groups (*F*_(2.311, 83.201)_ = 17.224, *p* < 0.001, *η*_p_^2^ = 0.324); however, no interaction emerged between training scores and age group, yielding no differences in the visual training manipulation among young and older adults (*F*_(2.311, 83.201)_ = 1.376, *p* = 0.258, *η*_p_^2^ = 0.037). Average response accuracy appeared to improve across the four days of training for both age groups (young adults: *F*_(1.799, 32.376)_ = 5.846, *p* = 0.008, *η*_p_^2^ = 0.245; older adults: *F*_(3, 54)_ = 11.856, *p* < 0.001, *η*_p_^2^ = 0.397), indicating that participants were consistently able to recognize movements that had appeared in the visually trained videos. This effect was driven by a significant improvement from day 1 to day 2 (young adults: *t*_(18)_ = −2.851, *p* = 0.011; older adults: *t*_(18)_ = −4.237, *p* < 0.001) and no further significant improvements between any other pairs of consecutive days (all *p* values > 0.05: young adults: day 2 versus day 3: *t*_(18)_ = −0.498, *p* = 0.624; day 3 versus day 4: *t*_(19)_ = −1.299, *p* = 0.210; older adults: day 2 versus day 3: *t*_(18)_ = 0, *p* = 1; day 3 versus day 4: *t*_(18)_ = −0.137, *p* = 0.893). This suggests that the performance level of participants in both age groups reached ceiling after the second day of training.

#### 3.1.2. Physical Performance on Day 5

On the final day of testing, after the second fMRI session was completed, participants were asked to perform all six dance sequences (two from each training category: physically trained, visually trained, and untrained conditions) in order to generate an objective measure of their ability to perform each of the dance movements they observed during both fMRI sessions and assess the impact of differentiated sensorimotor experience on motor performance (Figure 2(c)).

A main effect of training type was observed across groups (*F*_(1.684, 62.307)_ = 46.048, *p* < 0.001, *η*_p_^2^ = 0.554) as well as an interaction between age group and training condition (*F*_(1.684, 62.307)_ = 8.139, *p* = 0.001, *η*_p_^2^ = 0.180), and a main effect of age confirming that young adults performed better overall than older adults (*F*_(1, 37)_ = 15.013, *p* < 0.001, *η*_p_^2^ = 0.289). A one-way ANOVA ran within each age group separately revealed a main effect of training type on dance performance in both groups (young adults: *F*_(2, 38)_ = 40.802, *p* < 0.001, *η*_p_^2^ = 0.682; older adults: *F*_(2, 36)_ = 9.284, *p* = 0.001, *η*_p_^2^ = 0.340). Pairwise comparisons indicate that physically trained sequences were performed significantly better than visually trained or untrained sequences, among both young and older adults (young adults: physically versus visually trained: *t*_(19)_ = 7.556, *p* < 0.001; physically trained versus untrained: *t*_(19)_ = 7.251, *p* < 0.001; older adults: physically versus visually trained: *t*_(18)_ = 3.275, *p* = 0.008; physically trained versus untrained: *t*_(18)_ = 3.551, *p* = 0.004). Differences in performance between observed and untrained sequences did not reach significance, with the Bonferroni-corrected alpha level of 0.025 (young adults: *t*_(19)_ = 2.331, *p* = 0.031; older adults: *t*_(18)_ = 1.613, *p* = 0.124).

#### 3.1.3. Training Modality Categorization Task Performance

As a follow-up task, immediately following the post-training scan on day 5 of the study, participants were asked to watch each silhouette stimulus again (6 per training condition) and to categorize each stimulus as either being physically trained, visually trained, or untrained (see Supplementary Figure 1). Overall, young adults were better than older adults in identifying the training category to which each stimulus belonged (*F*_(1, 36)_ = 65.560, *p* < 0.001). Moreover, we observed a main effect of the training type (*F*_(2, 56.315)_ = 25.501, *p* < 0.001) and a nonsignificant trend towards an interaction between training category and age group (*F*_(2, 56.315)_ = 3.093, *p* = 0.065). This marginal interaction suggests that older adults are overall not performing poorly at recognizing the training category for those videos that have been physically or visually trained (all above chance level). However, they do perform particularly poorly at categorizing the untrained videos (categorizing them as trained in 79% of cases). It is to note that this task was not designed to definitively determine how well participants can match specific movements with a prior training condition (as was the aim in [48]), as participants only performed a total of 18 trials in this follow-up test (one trial for each stimulus). However, this exploratory follow-up task was designed to give us an indication of how well young and older adults can explicitly categorize the training condition associated with the different moves they observed in the scanner.

### 3.2. Effect of Sensorimotor Training among Young and Older Adults at the Whole Brain Level

Our first neuroimaging objective was to examine the effects of physical and visual training on whole brain activity. We did this first within each age group separately and then comparing young with older adults. As a reminder, we always evaluated contrasts to explore both increases and decreases in BOLD responses after training (comparing post and pre-training scans in both directions).

All of the following analyses were run with both days modelled in the same design matrix at the 1st level (and preprocessed together). All contrasts were evaluated at *p*_unc_ < 0.001, *k* = 10 voxels, and here, we focus on those results that survive correction of *p*_FWEcorr_ < 0.05 at the cluster level.

#### 3.2.1. Brain Regions Involved in Physical and Visual Learning in Younger Adults

*(1) Effects of Physical Training: Younger Adults*. Among young adults, the observation of body movements after physical training compared to before (post-training > pre-training) did not yield any significant increases in neural activity. However, this same contrast did reveal several brain regions that demonstrated a decreased response after training, including areas associated with sensorimotor processing, such as the left paracentral lobule and postcentral gyrus and right precentral gyrus and inferior temporal gyrus. In addition, the amygdala and middle occipital gyrus also demonstrated decreased response amplitude when observing those movements that had been physically trained (Table 1(a)).

*(2) Effects of Visual Training: Younger Adults*. Among younger adults, the observation of body movements encountered after visual training compared to before did not lead to any significant increases in activation. In comparison, several brain regions in the right hemisphere showed a marked decrease in response amplitude after four days of visual training, including the fusiform gyrus, postcentral gyrus, and middle temporal gyrus (Table 1(b)).

#### 3.2.2. Brain Regions Involved in Physical and Visual Learning in Older Adults

*(1) Physical Training: Older Adults*. Among older adults, the observation of body movements after physical training compared to before physical training resulted in increased activation in the right precuneus, and activity decreases within the right superior and inferior parietal lobules (SPL and IPL) and the right thalamus (Table 2(a)).

*(2) Visual Training: Older Adults*. In older adults, the observation of body movements after visual training compared to before four days of visual training resulted in an increase of activation of the right precuneus, similarly than after physical training but with no significant decrease of activity in any region (Table 2(b)).

#### 3.2.3. Differences and Similarities between Age Groups, by Training Experience

**Figure 3:**
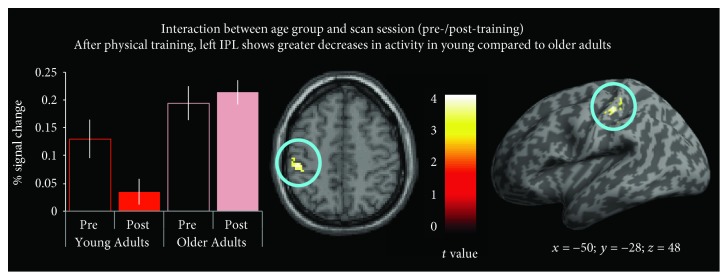
Training experience by age group interaction. The left inferior parietal lobule (IPL) demonstrates more marked decreases in engagement after physical training among the young adult participant sample compared to the older adults (*p*_FWE−corr_ = 0.01, *t* = 4.3).

*(1) Differences between Young and Older Adults*. In order to evaluate regions in which activation varied as a function of the age group, young and older adults were compared to each other while the factor performance gain was included as covariate of no interest. These contrasts were evaluated as two-sample *t*-tests. Compared to older adults, young adults showed decreased recruitment of the left inferior parietal lobule after training compared to before physical training (Figure 3). No other contrasts yielded any significant difference between young and older adults when comparing pre- and post-training scans (including the positive effect of performance). It is to note that running the same contrasts with performance on day 5 as the covariate of no interest (instead of performance gain) lead to a nearly identical pattern of activation, with the only significant activation emerging within the left inferior parietal lobule (*x* = −50, *y* = −28, *z* = 48, *p*_FWE−corr_ = 0.001, *t* = 4.8) when comparing young adults to older adults.

**Figure 4:**
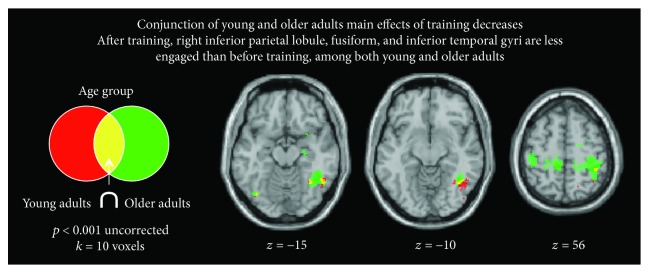
Common regions among young and older adults demonstrating a decreased response after physical training.

*(2) Common Regions Influenced by Training among Young and Older Adults*. When analysed separately, training effects among young and older adults mainly resulted in decreased activity after physical training compared to before. For this reason, we focused on this contrast to see which regions were similarly showing a decrease of activity in both age groups after physical training compared to before. This conjunction analysis (using *t* > 3.5 as the threshold) yielded overlapping activation across 87 voxels, in three main regions, namely, the left fusiform gyrus, the right inferior temporal gyrus, and the right inferior parietal lobule (Figure 4).

### 3.3. Effects of Training on Neural Representations of Actions

#### 3.3.1. Regions That Discriminate Better between Dance Movements after Compared to before Training in Each Age Group

This first set of decoding analyses is aimed at identifying which brain regions were better able to distinguish between physically trained and untrained movement sequences after compared to before training, among young and older adults separately.

All results reported are *p*_FWEcorr_ < 0.05 at the cluster level, with a threshold at *p*_unc_ < 0.005, *k* = 20 voxels.

**Figure 5:**
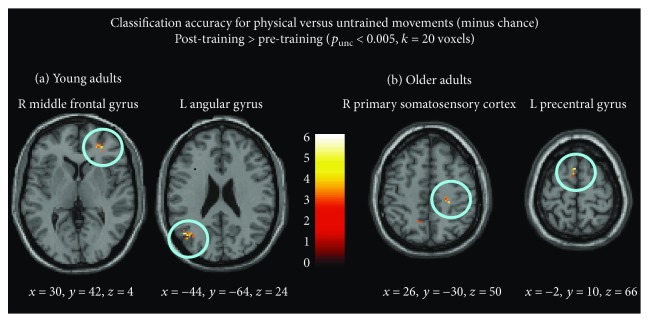
Regions that can accurately discriminate better between physically trained and untrained movements after training compared to before training. (a) In young adults. (b) In older adults. Coordinates of the centre of the cluster are given for all cluster-corrected regions (*x*, *y*, and *z* in MNI coordinates).

*(1) Younger Adults*. When comparing regions that could better distinguish between physically trained and untrained sequences after training compared to before training, the left angular gyrus (IPC) and right middle frontal gyrus (close to IFS) were the two only regions that could discriminate accurately (i.e., above chance) after training between physically trained and untrained sequences (Figure 5(a) and Table 3(a)).

*(2) Older Adults*. When comparing regions that could distinguish between physically trained and untrained sequences after training better than before training, left SMA and primary somatosensory cortex were the only regions that came close to the FWEcorr threshold criteria (*p*_FWEcorr_ = 0.05 and *p*_FWEcorr_ = 0.07, resp.; Figure 5(b) and Table 3(b)).

#### 3.3.2. Effect of Age on Discrimination Accuracy after Physical Training

**Figure 6:**
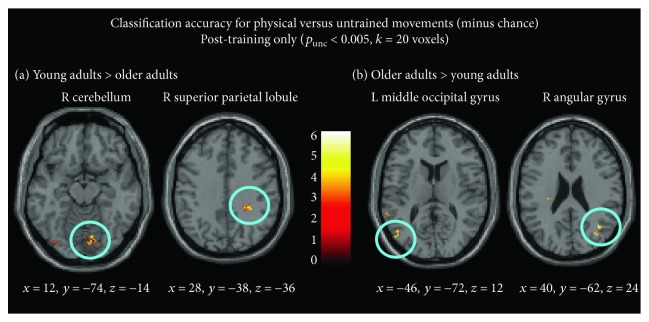
Direct comparison of classification accuracy for physical versus untrained sequences, minus chance, comparing young and older adults. (a) Brain regions that are better able to discriminate between physical and untrained movements in young adults compared to older adults. (b) Brain regions better able to discriminate between physical and untrained movements in older adults compared to young adults. Coordinates are reported in MNI space (*x*, *y*, and *z*).

In the following analyses, only accuracy maps from post-training scans were taken into account, to explore which regions can accurately discriminate between physically trained sequences and untrained sequences after training, comparing younger and older adults. When evaluating this contrast on the post-training data, the right superior parietal lobule and the right cerebellum discriminate between physically trained and untrained sequences more accurately in young adults compared to older adults (Figure 6(a) and Table 4(a)). In contrast, the inverse contrast, evaluating brain regions that discriminate between physically trained and untrained sequences better in older adults compared to young adults, yielded the right angular gyrus and left middle occipital gyrus (Figure 6(b) and Table 4(b)).

**Figure 7:**
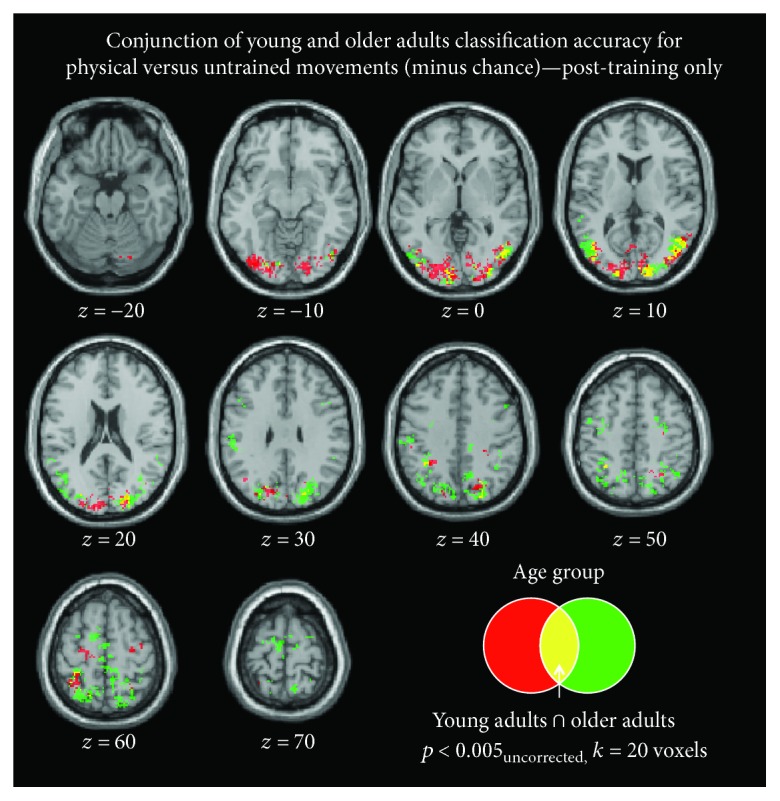
Conjunction analysis: common regions among young and older adults for accurately discriminating between physical and untrained sequences, on day 5.

Finally, we conducted an exploratory conjunction analysis on the accuracy maps (using *t* > 3.5 as the threshold), to determine whether there were any common brain regions among young and older adults that could distinguish between physically trained and untrained sequences after training (for full results of these maps on day 5 for young and older adults separately, please see Supplementary Materials). This conjunction analysis yielded overlap across 794 voxels, including multiple subregions within the occipital cortex, as well as sensorimotor areas, including the left paracentral gyrus (Figure 7).

## 4. Discussion

In the present study, we manipulated the type of sensorimotor experience that young and older adults received with dance sequences to determine how experience shapes responses at behavioural and brain levels, during early and later adulthood. We set out to address three main questions: (i) How do different types of training influence complex action performance among young compared to older adults? (ii) How do different types of training shape brain responses at a global level during action observation among young compared to older adults? (iii) How does physical training shape brain responses at the level of action representations?

Overall, participants' behavioural performance on motor and visual tasks improved across the training period, with younger adults showing greater performance gains than older adults. At the brain level, both age groups demonstrated overall *decreases* in sensorimotor cortical engagement after physical training, with younger adults showing more pronounced decreases in the inferior parietal lobule (IPL) activity compared to older adults. However, it is of note that the older adult population also showed an *increase* in precuneus activity after training. Neural decoding results show that among both young and older adults, visual and motor cortices contain experience-specific representations of new motor learning. In the following, we consider each of these findings, including how they relate to prior work and how they might form a foundation for future research.

### 4.1. Behavioural Differences between Younger and Older Adults

Both young and older adults' performance on both behavioural tasks (physical and visual learning tasks) improved across the four days of training. However, young adults outperformed older adults overall in terms of physical performance, and as Figure 2(a) shows, this performance discrepancy was present from the first day of training. This pattern of findings is likely explained by overall better physical condition and motor learning abilities among younger adults, whose movements are also more fluid than those made by individuals in advanced age [46, 63, 64]. However, the young adults did not outperform older adults in terms of recognition accuracy on the visual training task. This might be driven by the fact that participants in both age groups found the recognition task very easy, and the plots of behavioural performance on this task (Figure 2(b)) document that participants from both age groups were more or less performing at ceiling from day 2 onwards.

When considering physical performance score discrepancies between young and older adults, it is also worth noting that the Kinect motion tracking system and the Xbox scoring system take into account the fluidity or smoothness of a performer's movements and participants have the potential to score many extra “bonus points” when they reproduce a move being performed by the avatar on screen in a particularly smooth manner. This aspect of the video game design might have penalized older adults' physical performance scores, as research demonstrates that gross bodily movements become less fluid/smooth with advancing age [46]. However, such differences between young and older adults' general kinematics should not affect the overall improvements across days within each age group, but here again, we also see more marked improvements among younger adults. To better understand the underlying neural basis for these differences between the two age groups, we investigated our main hypotheses by means of fMRI.

### 4.2. Effect of Training at a Global Brain Level during Action Observation

Taken together, the univariate analyses show a similar pattern of results among both young and older adults, characterised by activity decreases post-training. Specifically, evidence emerges that the right ITG and IPL, brain regions associated with the AON, show decreased response amplitude after physical training among both groups of participants. The left fusiform gyrus demonstrates a similar response profile in both groups. It thus appears that, regardless of age, physical training results in less recruitment of these sensorimotor and higher-order visual association cortices that are robustly recruited when observing unfamiliar or novel actions [26, 27, 65]. In a recent meta-analysis, Hardwick and colleagues [34] report similar decreases in activity among these same brain regions following motor learning, even though many of the studies included in this meta-analysis report increases in activity after training. This line of results helps to further illuminate how physical training influences brain regions recruited during observation of complex whole-body movements, with no other cue than a pared-down silhouetted representation of the movement.

The only increase of activity found after training in the present study emerged from the older adults group in the right precuneus, after both physical and visual training. This finding suggests that in advanced age, the precuneus is sensitive to the type of visuomotor training examined in the present study. More generally, the precuneus has been implicated in higher-order cognitive processes such as episodic memory, motor imagery, and spatial aspects of motor behaviour control [66–68] and has also been shown to respond more robustly during motor tasks in advanced age [69]. However, as this region did not emerge from the direct contrast comparing older and younger adults, it is not possible to conclude that its increased engagement when observing movements that have been associated with visuomotor or visual experience is due to age, per se. Further investigation, perhaps taking a longitudinal approach, would be beneficial for clarifying the relationship between precuneus activity, aging, and visuomotor learning.

The fact that we fail to find robust increases of activity within brain regions classically associated with the AON after physical training, as we report previously with similar training paradigm [3, 18], might seem surprising at first, until a number of factors are taken into consideration. First, the pattern of results we report here is broadly consistent with the fine movement motor learning literature [39, 59], which reliably documents decreases in whole brain activity after training as a signature of neural efficiency [17, 37, 70, 71]. For example, Higuchi and colleagues [71] report that for both observation and execution of guitar chords, reliable neural efficiency effects emerged across training days. The field would benefit from studies using complex, multisensory training paradigms looking more systematically at both increases and decreases of activity. A recent study by Gardner and colleagues has attempted to explore the issue of post-training increases and decreases in neural response amplitude by using guitar training to probe the subjective nature of the prediction error signal [17]. These authors report results consistent with a predictive coding account of AON engagement during action observation and execution that also takes into account effects of changes in neural efficiency, providing a promising theoretical grounding for taking this work further.

It is also important to consider how the training and testing approach used in the present study differs from prior work that reports increased response amplitude with increased experience (e.g., [5, 6, 19, 22, 49]). First, many prior studies look at effects of years, if not decades, of training experience on perception [5, 6, 22], while those examining the effects of aging often study how physical skills that older adults learned when they were still young are perceived [43, 49]. Such a short-term training intervention, like the one used in the present study, might thus be suboptimal for generating robust age-related differences in brain activity. In addition, the movements observed during brain scanning were far simpler in the present study than those used in many previous studies [6, 18, 19, 22, 53]. As such, the kind of learning examined in the present study might be quite different from what our group and others have previously found [18, 49]. In further contrast to previous studies, the pared-down action silhouettes used here revealed no further information about each action beyond its kinematics [48]. This means that any training effect found at the brain level should be linked to pure motor learning and visual movement processing, which might further contribute to subtle findings compared to previous work in this vein. Moreover, in contrast to recent work by our group [18], it is important to note that the task participants performed in the scanner was also markedly different and very likely contributed to the different pattern of results we report here. In Kirsch and Cross's earlier study [18], each trial was followed by one of two questions: “How much did you *like* the movement you just watched?” and “How well could you *reproduce* the movement you just watched?” It should be evident that these two questions required much deeper kinematic, visual, and aesthetic processing than what was required in the present study (participants watch closely each movement and respond to occasional probe questions about very simple kinematic features of the previously viewed movement). For this reason, it is imperative to consider the different tasks and stimuli when evaluating discrepant results reported by previous studies that have used similar training paradigms, such as Kirsch and Cross [18], and the present study. These different patterns of findings also provide a useful consideration for future studies, namely, future work can examine the extent to which decreases in sensorimotor engagement following training are due to strengthened purely ‘kinematic' representations or to observers' not being prompted to think about their physical abilities to reproduce observed moves during action observation.

Even though the right IPL was found to be less activated after training among both age groups (Figure 4), the left IPL was the only region to demonstrate more marked decreases in engagement after physical training among young compared to older adults (Figure 3). The parameter estimate plot on this figure suggests that this finding is being driven by decrease in IPL engagement among young adults, while this region's response profile remains relatively unchanged among older adults. This could be indicative of processing efficiency gains among young adults that are simply not present among older adults. It is to note that no regions showed a greater decrease or increase after training in older adults compared to younger adults, when we control for physical gain performance. The IPL is one of the core regions of the AON, and its activity has been shown to be modulated by training [6, 16, 18].

In terms of visual training effects, we observed decreased engagement of sensorimotor and visual processing regions among young adults, similar to physical training. This is in line with previous literature showing common regions sensitive to observational and physical training [1–3, 14, 18]. However, among older adults, no regions showed decreased activity after visual training. This raises the possibility that visual training effects among older adults are either subtler or perhaps altogether different, perhaps due to declines in attentional and visual processing decline with advancing age [34]. A challenge for future research will be to examine in more depth age-related differences in observational training effects, possibly using a different task that can better capture performance and improvement via observation (which our task clearly failed to capture, as all participants were performing at ceiling by day 2).

Moreover, taking into account the results from the training modality categorization task participants completed after the post-training scan session, one could argue that the differences seen between young and older adults' brain activity is due to older adults not being able to accurately identify the training modality of the observed movements. Our results show that older adults performed more poorly than young adults on this task overall and that they were more likely to categorize untrained moves as trained. This finding of a carry-over effect is consistent with previous studies showing that older adults frequently show this tendency to misclassify new material as being familiar, which has been interpreted as showing that internal representations of events become more rigid with age [72]. However, as this categorization task did not include multiple trials for each stimulus (thus reducing its power) and our main aim was to examine differences before and after training within one training category (physical practice), we are reluctant to place too much stock in age differences on this task in our particular study. One final reason to be cautious with how we interpret young and older adults' performance on the categorization task is because even if older adults do not explicitly recognize the training category to which each stimulus belongs as well as younger adults, our main interest was in the neural implementation of the training, where we do see differences.

### 4.3. Examining the Impact of Physical Training on a Representational Level

The present study was designed to enable investigation of how physical training shapes brain responses not just at the level of magnitude differences but also at the level of more fine-grained action representations. The aim of our first set of pattern analyses was to identify brain regions that can distinguish between physical and untrained movement sequences after training compared to pre-training, among young and older adults separately. We predicted this between-category classification accuracy to be better overall after training compared to before training, when all stimuli were equally unfamiliar. Among younger adults, we found that the left angular gyrus and MFG could discriminate better after training between physically trained and untrained sequences, whereas among older adults, the left supplementary motor area and sensorimotor cortex could discriminate better after training between physically trained and untrained sequences.

The second set of analyses compared classification accuracies between the age groups, in particular their accuracy at distinguishing between physically trained and untrained sequence after physical training. These analyses yielded clear age-related differences, with better discrimination within the right superior parietal lobule and cerebellum in young compared to that in older adults. In contrast, older adults showed better discrimination in visual processing areas and multisensory integration areas such as the right angular gyrus and left MOG, compared to young adults.

This broad pattern of results is consistent with literature documenting that older adults rely more on sensory cortices during action observation than younger adults, even if the observed actions are familiar to them [47]. Computational models of cognitive aging posit that neural representations become less distinctive in old age [73]. Recent work by Carp and colleagues [74] reported that neural distinctiveness was reduced in older adults throughout the motor network. Neuroimaging studies of visual perception support this view, indicating that distributed patterns of brain activation evoked by different visual stimuli are less distinctive among older adults compared to young adults [75, 76]. In the present study, our findings do not speak to reductions in neural distinctiveness in advanced age per se, but instead we find different regions involved in distinguishing between different categories of stimuli (physically trained versus untrained) in young compared to older adults. As such, our findings suggest that the kind of training manipulation we used here has a specific effect that goes beyond the natural dedifferentiation that occurs with aging and instead leads to distinct networks mediating training-induced neural representations among young compared to older adults. While this part of the study was exploratory in nature, it provides useful point of departure for continued investigation of the changing nature of the representation of new sensorimotor learning across the lifespan. Moreover, one might speculate that older adults process the stimuli differently than younger adults or are at a different stage of learning when being scanned (as the behavioural data might suggest). These factors could also explain some of the differences seen with young adults. Older adults might still be in the consolidation phase on day 5, whereas in young adults, the top-down modulation of sensory regions has possibly already taken place [45, 47, 77]. Future studies could add scanning sessions at more time points (or include longer training manipulations with scanning sessions interspersed regularly throughout them), in order to explore whether older adults achieve the same performance as young adults, which is also reflected as (more) similar patterns of brain activation.

Finally, we explored which brain regions among young and older adults similarly distinguish between physically trained and untrained movements post-training. This conjunction analysis yielded common regions in occipital and motor cortices, demonstrating finer motor representations on day 5 for both age groups. This suggests that physical training plays an important role in the coding of fine movements in higher-order visual and motor areas, lending further support to the neural efficiency theory and findings in the motor domain [39].

### 4.4. Limitations and Future Directions

As with any study and with exploratory studies in particular, this work has several important limitations and has raised a number of possibilities for future research that warrant consideration. Since the present study was the first to tackle questions of complex action learning at brain and behavioural levels among young and older adults, we chose to investigate age group differences when both groups attempt to learn initially novel actions for the same amount of time. In this way, performance at the end of training was consequently not matched between the age groups. Thus, we cannot clearly disentangle whether our between-age group results are related to the way participants learn and represent dance sequences or due to the fact that young and older adults are at different stages of the learning process. An alternative design could have continued training the older adults until they performed at the same level as the younger adults and then compared brain activity. Future studies might explore this possibility and implement paradigms in which performance is better matched at the end of training. However, with respect to physically trained sequences in particular, general changes in motor control might prevent older adults from reaching the same performance level as younger adults irrespective of the amount of training provided [63].

Moreover, the technology used for our training intervention might have had an impact on participants' dance experience and the extent to which they perceive the task as “real” dancing. Similarly, age differences could possibly impact participants' willingness to dance in front of a large TV with avatars. However, devices such as the Kinect system have become more and more common in many households in recent years. Consequently, more on more people, including older generations, are becoming accustomed to interacting with them (and indeed, several of our older participants mentioned at the conclusion of the study that they had seen their grandchildren playing games with the Xbox Kinect in the past and had never participated as they thought the technology was not for them, but their minds had been changed since taking part in our study). In addition, we asked older adults to try the Kinect dance set up in a separate session before actual testing, and only those older adults who felt comfortable to do the training took part in the main experiment. Finally, as all participants also reported having enjoyed the experience, we believe that our results are not biased due to differences in the perception of the training set-up between young and older adults.

## 5. Conclusions

To our knowledge, this is the first study to combine a complex, whole-body training paradigm with univariate and multivariate analyses to investigate the impact of sensorimotor learning on action representations among young and older adults. While this study was exploratory in nature, our results should contribute to building a more complete picture of age-related changes in experience-dependent plasticity. Ultimately, we hope that insights gained from this approach will inform visuomotor learning and rehabilitation interventions for those in early and advanced adulthood.

## Figures and Tables

**Table:** (a) Physical training

Region	BA	MNI coordinates			*t* value	Cluster size	*p*FWE_corr_ value
		*x*	*y*	*z*			
*(i) Increase post-training compared to pre-training*							
No cluster survived the threshold							
*(ii) Decrease post-training compared to pre-training*							
**L paracentral lobule**	**5**	**−10**	**−34**	**58**	**7.17**	**233**	**<0.001**
L postcentral gyrus	1	−28	−36	56	4.50		
L superior parietal lobule	3b	−20	−42	58	4.13		
**R precentral gyrus**	**4**	**34**	**−28**	**58**	**6.02**	**706**	**<0.001**
R postcentral gyrus	1	38	−38	56	5.07		
R postcentral gyrus	1	26	−32	58	5.06		
**R inferior temporal gyrus**	**37**	**50**	**−50**	**−16**	**5.89**	**380**	**<0.001**
R inferior temporal gyrus	37	40	−52	−14	5.51		
R inferior temporal gyrus		36	−60	−4	3.85		
**R hippocampus**		**26**	**−4**	**−24**	**5.65**	**168**	**0.003**
R hippocampus		34	−4	−22	5.05		
R middle temporal gyrus	21	46	−4	−20	4.55		
**L postcentral gyrus**	1	**−40**	**−30**	**56**	**5.00**	**94**	**0.048**
L postcentral gyrus	1	−44	−22	56	4.26		
**R middle occipital gyrus**	**19**	**46**	**−76**	**18**	**4.63**	**153**	**0.005**
R middle occipital gyrus	19	40	−84	14	4.59		
R middle occipital gyrus	19	52	−64	14	4.55		

**Table:** (b) Visual training

Region	BA	MNI coordinates			*t* value	Cluster size	*p*FWE_corr_ value
		*x*	*y*	*z*			
*(i) Increase post-training compared to pre-training*							
No cluster survived the threshold							
*(ii) Decrease post-training compared to pre-training*							
L inferior temporal gyrus	20	−40	−32	−18	6.30	83	0.081
L inferior temporal gyrus	37	−42	−40	−18	4.68		
L fusiform gyrus	37	−38	−56	−18	4.14		
R amygdala		30	−2	−24	5.68	80	0.092
R parahippocampal gyrus	36	24	−26	−24	4.44		
R parahippocampal gyrus	36	20	−12	−24	4.36		
**R fusiform gyrus**	**37**	**42**	**−42**	**−16**	**5.58**	**394**	**<0.001**
R inferior temporal gyrus	37	40	−52	−14	4.86		
R inferior temporal gyrus	37	50	−50	−16	4.46		
**R postcentral gyrus**	**1**	**30**	**−32**	**58**	**5.40**	**498**	**<0.001**
R postcentral gyrus	1	28	−40	66	4.86		
R postcentral gyrus	1	36	−34	66	4.69		
**R middle temporal gyrus**	**19**	**52**	**−66**	**12**	**5.36**	**180**	**0.002**
R middle occipital gyrus	19	46	−80	12	4.88		
R middle occipital gyrus	39	40	−58	12	3.91		

BA: Brodmann's area; R: right; L: left. Analysis performed at *p* < 0.001, uncorrected, *k* = 10 voxels, and regions in bold font are FWE cluster corrected at the *p* < 0.05 level. Up to three local maxima are listed when a cluster has multiple peaks more than 8 mm apart.

**Table:** (a) Physical training

Region	BA	MNI coordinates			*t* value	Cluster size	*p*FWEcorr value
		*x*	*y*	*z*			
*(i) Increase post-training compared to pre-training*							
**R precuneus**	**7**	**4**	**−68**	**30**	**6.68**	**510**	**<0.001**
R precuneus	31	10	−66	24	5.45		
L cuneus	18	−6	−66	22	4.98		
*(ii) Decrease post-training compared to pre-training*							
**R thalamus**		**8**	**−26**	**6**	**5.88**	**314**	**<0.001**
L thalamus		−2	−24	6	5.66		
L thalamus		−12	−32	2	5.26		
**R superior parietal lobule**	**39**	**32**	**−56**	**48**	**5.55**	**187**	**0.002**
R superior occipital gyrus	7	22	−62	48	4.72		
R superior parietal lobule	7	26	−50	46	4.31		
R inferior temporal gyrus	37	50	−50	14	5.22	69	0.147
R inferior temporal gyrus		44	−54	−6	4.53		
R fusiform gyrus		34	−54	−12	3.97		
**R inferior parietal lobule**	**1**	**60**	**−20**	**36**	**4.92**	**182**	**0.002**
R supramarginal gyrus	40	60	−30	38	4.83		
R supramarginal gyrus	40	54	−28	48	4.05		

**Table:** (b) Visual training

Region	BA	MNI coordinates			*t* value	Cluster size	*p*FWEcorr value
		*x*	*y*	*z*			
*(i) Increase post-training compared to pre-training*							
**R precuneus**	**31**	**10**	**−66**	**24**	**5.67**	**220**	**<0.001**
R precuneus	31	18	−62	22	4.34		
L precuneus	31	−2	−66	28	4.33		
*(ii) Decrease post-training compared to pre-training*							
No cluster survived the threshold (all *p*FWEcorr > 0.1)							

BA: Brodmann's area; R: right, L: left. *p* < 0.001, uncorrected; *k* = 10 voxels. Regions in bold font are FWE cluster corrected at the *p* < 0.05 level. Up to three local maxima are listed when a cluster has multiple peaks more than 8 mm apart.

**Table 3:** Regions that can accurately discriminate better between physically trained and untrained movements after training compared to before training. (a) Young adults. (b) Older adults.

Region	BA	MNI coordinates			*t* value	Cluster size	*p*FWEcorr value
		*x*	*y*	*z*			
*(a) Physically trained versus untrained in younger adults*							
**L angular gyrus**	**39**	**−44**	**−64**	**24**	**6.05**	**69**	**<0.001**
L angular gyrus	39	−34	−66	26	4.00		
L middle occipital gyrus	19	−38	−68	18	3.94		
**R middle frontal gyrus**	**10**	**30**	**42**	**4**	**4.68**	**40**	**0.048**
R middle frontal gyrus	10	28	48	−2	4.21		
R middle frontal gyrus		28	40	12	3.35		
*(b) Physically trained versus untrained in older adults*							
R postcentral gyrus	3	26	−30	50	6.12	46	0.053
R middle cingulate cortex		16	−28	46	4.38		
L precentral gyrus	6	−2	10	66	5.68	43	0.078
L precentral gyrus	6	−8	2	60	3.75		

BA: Brodmann's area; R: right, L: left. *p* < 0.001, uncorrected; *k* = 10 voxels. Regions in bold font are FWE cluster corrected at the *p* < 0.05 level. Up to three local maxima are listed when a cluster has multiple peaks more than 8 mm apart.

**Table 4:** Physical versus untrained classification accuracy minus chance comparing young and older adults. (a) Young adults > older adults. (b) Older adults > young adults.

Region	BA	MNI coordinates			*t* value	Cluster size	*p*FWEcorr value
		*x*	*y*	*z*			
*(a) Young adults > older adults*							
**R superior parietal lobule**		**28**	**−38**	**36**	**4.62**	**46**	**0.039**
**R cerebellum**		**12**	**−74**	**−14**	**4.43**	**81**	**<0.001**
R calcarine gyrus	18	14	−70	−6	4.35		
R lingual gyrus	17	8	−82	−12	3.86		
*(b) Older adults > young adults*							
**R angular gyrus**	**39**	**40**	**−62**	**24**	**4.28**	**46**	**0.039**
R middle occipital gyrus		34	−70	26	4.07		
**L middle occipital gyrus**	**19**	**−46**	**−72**	**12**	**4.24**	**47**	**0.034**
L middle temporal gyrus	39	−50	−62	18	4.15		
L middle temporal gyrus	39	−40	−60	20	3.62		

BA: Brodmann's area; R: right, L: left. *p* < 0.001, uncorrected; *k* = 10 voxels. Regions in bold font are FWE cluster corrected at the *p* < 0.05 level. Up to three local maxima are listed when a cluster has multiple peaks more than 8 mm apart.

## Supplementary Materials

Supplementary Materials Supplementary Table 1: regions that can accurately discriminate better between physically trained and untrained movements on day 5. (a) Younger adults. (b) Older adults. Supplementary Figure 1: recognition accuracy average scores in young and older adults. After the post-training scan on day 5, participants watched each dance movie stimulus again (6 training conditions) and were asked to categorize them as either being physically trained, visually trained, or untrained.
