# Supplementary material for: Dance Training Shapes Action Perception and Its Neural Implementation within the Young and Older Adult Brain
Source: Neural Plast. 2018 Jul 12;2018:5459106. doi: 10.1155/2018/5459106 (PMC6079376; doi:10.1155/2018/5459106)
Supplement: Supplementary Materials — Supplementary Table 1: regions that can accurately discriminate better between physically trained and untrained movements on day 5. (a) Younger adults. (b) Older adults. Supplementary Figure 1: recognition accuracy average scores in young and older adults. After the post-training scan on day 5, participants watched each dance movie stimulus again (6 training conditions) and were asked to categorize them as either being physically trained, visually trained, or untrained. [file 5459106.f1.pdf]

Supplementary Materials

Supplementary Table. Regions that can accurately discriminate better between physically trained and untrained movements on Day 5. (a). Younger Adults. (b). Older Adults.

Region	BA	MNI Coordinates x	y	z	T-value	Cluster Size	P FWE$_{corr.}$ Value
(a) Physically trained vs Untrained in Younger Adults on Day 5							
**L Inferior Occipital Gyrus**	**19**	**-34**	**-80**	**-4**	**8.11**	**7323**	**<0.001**
R Middle Occipital Gyrus	19	46	-72	4	7.96		
R Middle Temporal Gyrus	19	48	-62	14	7.47		
**L Inferior Parietal Lobule**	**7**	**-30**	**-46**	**40**	**6.29**	**580**	**<0.001**
L Inferior Parietal Lobule	39	-40	-54	54	5.67		
L Superior Parietal Lobule	7	-28	-48	68	5.48		
**R Superior Parietal Lobule**	**1**	**26**	**-34**	**40**	**5.94**	**72**	**0.001**
R Superior Parietal Lobule	1	32	-34	34	3.71		
R Superior Parietal Lobule	6	18	-38	40	3.57		
**L Paracentral Gyrus**	**6**	**-20**	**-14**	**56**	**5.67**	**408**	**<0.001**
R Supplementary Motor Area	6	6	-12	66	5.64		
L Paracentral Gyrus	6	-10	-20	64	5.21		
**R Superior Parietal Lobule**	**7**	**20**	**-56**	**48**	**5.48**	**313**	**<0.001**
R Paracentral Gyrus	1	4	-38	60	4.79		
R Superior Parietal Lobule	7	14	-54	66	4.77		
**R Precentral Gyrus**	**6**	**26**	**-16**	**62**	**5.48**	**274**	**<0.001**
R Precentral Gyrus	6	38	-12	60	4.95		
R Precentral Gyrus	6	30	-14	48	4.92		
**R Middle Frontal Gyrus**	**9**	**30**	**38**	**44**	**5.08**	**52**	**0.009**
R Middle Frontal Gyrus	9	30	32	50	4.40		
R Superior Frontal Gyrus	8	22	44	44	3.33		
**L Inferior Parietal Lobule**	**40**	**-52**	**-34**	**42**	**4.61**	**136**	**<0.001**
L Inferior Parietal Lobule	40	-44	-30	26	4.47		
L SupraMarginal Gyrus	40	-52	-24	34	4.40		
(b) Physically trained vs Untrained in Older Adults on Day 5							
**R Cuneus**	**19**	**16**	**-78**	**32**	**10.70**	**11863**	**<0.001**
R Middle Temporal Gyrus	19	52	-64	8	8.41		
R Superior Occipital Gyrus	19	20	-86	28	8.17		
**L SupraMarginal Gyrus**	**40**	**-54**	**-26**	**32**	**7.65**	**663**	**<0.001**
L Postcentral Gyrus	40	-48	-22	42	5.75		
L SupraMarginal Gyrus	40	-60	-26	38	4.93		
**L Inferior Frontal Gyrus**	**8**	**-48**	**18**	**32**	**7.32**	**141**	**<0.001**
L Inferior Frontal Gyrus	44	-42	24	24	5.86		
L Middle Frontal Gyrus		-34	18	32	4.07		
**R SupraMarginal Gyrus**	**40**	**52**	**-38**	**26**	**5.56**	**96**	**<0.001**
R Superior Temporal Gyrus	39	52	-46	20	5.21		
R Superior Temporal Gyrus	22	60	-38	14	4.17		
**R Inferior Frontal Gyrus**	**44**	**48**	**16**	**30**	**5.08**	**103**	**<0.001**
R Middle Frontal Gyrus	8	40	10	36	4.79		
R Inferior Frontal Gyrus	9	42	22	22	4.51		
**L Precentral Gyrus**	**6**	**-52**	**2**	**16**	**4.92**	**54**	**0.011**
L Rolandic Operculum	44	-44	4	14	3.79		
L Rolandic Operculum	6	-46	-4	14	3.06		

BA, Brodmann's area; R: right, L: left; $P < 0.005$, uncorrected; k=20voxels. Regions in bold font are FWE-cluster corrected at the $p < 0.05$ level. Up to three local maxima are listed when a cluster has multiple peaks more than 8 mm apart.

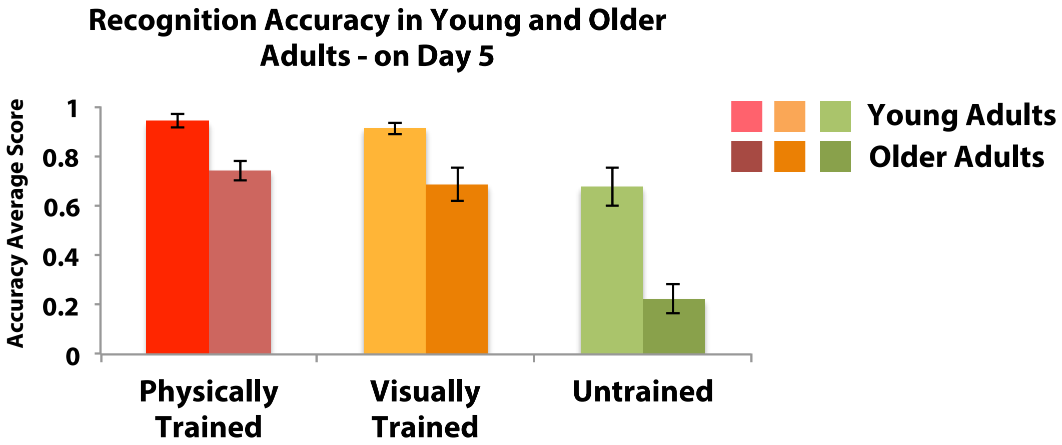

Supplementary Figure 1. Recognition Accuracy Average Scores in Young and Older Adults. After the post-training scan on Day 5, participants watched each dance movie stimulus again (6 per training condition) and were asked to categorize them as either being physically trained, visually trained or untrained.
